# Presence of SARS‐CoV‐2 in plural effusion with COVID‐19 patients

**DOI:** 10.1002/ccr3.4130

**Published:** 2021-05-15

**Authors:** Zahra Ataee, Sepideh Hasanzadeh, Ali Mehri, Mahdiyeh Sayadi, Leila Ataei, Saeid Amel Jamehdar

**Affiliations:** ^1^ Department of Internal Medicine School of Medicine Mashhad University of Medical Sciences Mashhad Iran; ^2^ Antimicrobial Resistance Research Centre Mashhad University of Medical Sciences Mashhad Iran; ^3^ Department of Microbiology and virology School of Medicine Mashhad University of Medical Sciences Mashhad Iran; ^4^ Student Research Committee Faculty of Medicine Mashhad University of Medical Sciences Iran

**Keywords:** plural effusion, Real‐time RT‐PCR, SARS‐CoV‐2

## Abstract

RT‐PCR of OP, NP swabs, is the gold standard test for the diagnosis of COVID‐19. CT scan plays an important role in the diagnostic evaluation of COVID‐19. Both methods increase the tracing disease. Even though pleural involvement has been reported less, it has been observed in significant cases.

## Graphical abstract

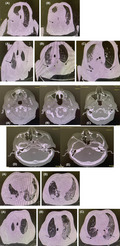

## 1 INTRODUCTION

Coronavirus disease 2019 (COVID‐19) is a pandemic infection caused by the severe acute respiratory syndrome virus SARS‐CoV‐2, resulting in a wide range of symptoms from mild to severe. The gold standard for diagnosis is nucleic acid detection by real‐time reverse transcriptase‐polymerase chain reaction in oropharyngeal swabs, nasopharyngeal washes, and nasal aspirates. However, due to the limitations in this technique's sensitivity, chest imaging, especially CT scan, plays a critical role in the diagnostic evaluation of COVID‐19. Although less common, pleural involvement has been mentioned in significant cases. This report describes the first cases of reverse transcriptase‐polymerase chain reaction detection of COVID‐19 in pleural fluid in Iran. Pleural effusion is not a common finding in COVID‐19 infection, but diagnosis in pleural effusion can be useful for optimizing diagnostic evaluation as well as patient management.

Coronavirus 2019 (COVID‐19) is a new coronavirus that has emerged in December 2019 in Wuhan and has a wide range of symptoms from mild to severe.^1^ The most common complaints of patients include fever (98%), dry cough (76%), dyspnea (55%), muscle pain, and fatigue (44%).^2^, ^3^ There is some evidence that gastrointestinal involvements, acute heart damage, and acute kidney damage are caused by COVID‐19.^4^, ^5^

Almost 19.3 million people have been infected with COVID 19 all over the world at the time this article has been written, August 8th, 2020. About 719 K deaths have been reported, and health systems face a threatening challenge worldwide. In Iran, 323 K people are infected with COVID 19, and 18,132 have been died due to the coronavirus.^6^ COVID‐19 is highly contagious, with an estimated mortality rate of 3.4%.^3^ Real‐time reverse transcriptase‐polymerase chain reaction (RT‐PCR) of oropharyngeal swabs, nasopharyngeal washes, and nasal aspirates is a gold standard test for diagnosis of COVID‐19.^7^ Sensitivity seems to be variable and affected by factors like selected “intrinsic” patient characteristics (eg, disease's stage and viral load) as well as technical aspects in collecting and managing specimens.^8^

Therefore, it should be decided more sensitively on the transfer of hospitalized patients who have been clinically diagnosed with COVID‐19.^9^ Chest imaging, especially CT scan, plays an important role in the diagnostic evaluation of COVID‐19. RT‐PCR and chest imaging methods increase the tracing disease in appropriate clinical settings and describe more atypical features as well as potential sampling targets (eg, pleural effusion).^10^ Although pleural involvement has been reported less, it has been observed in significant cases (pleural thickening, 32%, pleural effusion, 5%) and has been significantly associated with worse prognosis.^11^ To end, the SARS‐coronavirus‐2 epidemic, the diagnosis must be made quickly. The characteristics of pleural fluid in COVID‐19 have never been described in Iran. We describe the presence of SARS‐CoV‐2 in pleural effusion with COVID‐19 patients for the first time.

## 2 CASE REPORT

We present two cases of coronavirus disease 2019 (COVID‐19) admitted to the pulmonology unit of Imam‐Reza Hospital in Mashhad. All patients were male. One of them had a liver transplant and is still hospitalized at the time of submitting this manuscript. The other one was died within eight days after admission due to a myocardial infarction. The virus RNA was detected by real‐time RT‐PCR test in the pleural effusion of patients. Elevated C‐reactive protein (CRP), WBC, and creatinine were detected in all patients. Supportive care, antiviral, and antibiotic therapy were administered for all the patients according to GFR.

### 2.1 Case 1: A 69‐year‐old man

The patient has a history of hypertension and diabetes mellitus, with no history of smoking. He had dyspnea and cough two weeks before admission and complained of weight loss and anorexia on admission.

In the initial examination, the patient was not conscious, and the body temperature was 37.2°C, blood pressure 140/90 mm Hg, heart rate 82 beats/minute, respiratory rate 25/minute, and oxygen saturation of 75% on room air. The conjunctiva was slightly pale; also, on lung examination, tachypnea was observed, and a decrease of sound in the right lung and rules of the left lung was identified. Edema of both lower limbs up to the ankles was observed (+1). On general examination, no lymphadenopathy was observed, and other systems, including cardiac and abdominal examination, were normal.

On the day of admission, arterial blood gas (ABG) has shown the following results: pH = 7.36, PCO2 = 35 mm Hg, and HCO3 = 20 meq/L. Early laboratory tests discovered normal levels of blood glucose, electrolytes, calcium, phosphor, and magnesium. The urea and creatinine levels were 155 and 2.9, respectively. The blood sample revealed the following results: white blood cells count 14 300 cells per microliter with 4.7% lymphocytes and 90% neutrophils, mildly elevated C‐reactive protein (CRP = 82.2 mg/L). Depending on blood urea and creatinine levels, treatments were adjusted base on patient's GFR (glomerular filtration rate).

**FIGURE 1:**
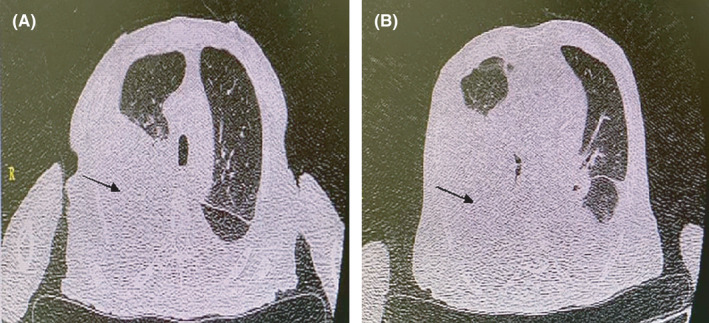
A‐B, HRCT chest imaging. A moderate volume of pleural effusion in the left lung, a large volume with a collapse of the lower lung on the right, and complete collapse of the lower right lobe of the right lung. A, On the third day of hospitalization and after three days of treatment. B, On the first day of hospitalization

During the hospitalization, blood investigation showed an increase in white cell count, lymphocyte dominance, relatively neutrophil decrease, and C‐reactive protein increase. Considering that the patient was an elderly man with Hb = 7.9, so on the first and second day of hospitalization, one unit of packed cell was transfused but due to repeated blood draws for biochemical tests and underlying disease, hemoglobin dropped again a little in next days. The further examinations included systemic CT demonstrating no evidence of brain edema. The first chest CT scan illustrated a moderate volume of pleural effusion in the left lung and a large volume with a collapse of the lower lung on the right (Figure 1). The complete collapse of the lower right lobe of the right lung and mediastinal and perivascular lymphadenopathy was observed (Figure 1). Due to the above findings, a chest tube was implanted on the right side.

**FIGURE 2:**
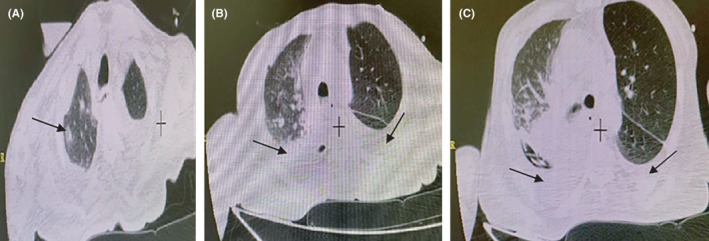
HRCT chest imaging. On the fourth day of hospitalization, and after implanting a chest tube. A, Patchy consolidation at the apex and centrilobular lung nodules on the right lung, B, moderate volume of pleural effusion in the left lung, and C, mildly volume hydropneumothorax with lower lung consolidation collapse on the right side after chest tube implantation

In the second chest CT, mildly volume pleural effusion was seen on the left side, and hydropneumothorax with lower lung consolidation collapse was seen on the right side after chest tube implantation. Besides, there was a patchy consolidation at the apex of the right lung (Figure 2).

During emergency treatment, endotracheal intubation and mechanical ventilation were required. The patient was started on antiviral therapy with Favipiravir 1600 mg bid on the first day and 600 mg bid for the next five days. Also, Remdesivir 200 mg was prescribed on the first day and 100 mg daily for the next 5 days. He was empirically started prophylactic antibiotic therapy (azithromycin 500 mg daily, vancomycin 1 gr every 48 hours, and dexamethasone 4 mg bid). He also subcutaneous administration of interferon and heparin (5000 units every 8 hours).

Moreover, he underwent intravenous administration of Meropenem 500mg bid and Meropenem dosage was adjusted according to the patient's GFR. The chest bottle chart was recorded daily. The results of all biochemical tests are available in Table 1. Based on chest radiographic results, 4 mL of clear yellow pleural fluid was removed, and the specimen was sent for differential macroscopic and microscopic analysis, cultures, biochemical examination, and SARS‐CoV‐2 RT‐PCR.

**TABLE 1:** The biochemical test during hospitalization

	1th day	2th day	3th day	4th day	5th day	6th day	7th day	8th day
WBC	14.3	16.3	15.6	13.3	14.2	14.8	13.6	13.5
Neutrophils	90%	96%	85%	82%	75%	80%	83%	83%
lymphocyte count	4.7%	2%	5.3%	7.6%	8.8%	7.8%	8%	8%
Hemoglobin	7.9	8.9	9.2	11.8	12.1	12.4	10.4	10
Platelet count	348	348	338	330	305	284	221	215
Urea	158	184	180	109	167	159	152	158
Creatinine	2.9	3	3.1	1.9	3	2.8	2.7	2.6
Total protein	5.1	5	5.2	4.9	5	5.1	5	5
Sodium	142	145	141	139	139	139	136	138
Potassium	4.1	4.9	4.9	5	4.8	4.4	4.5	4.6
O2 satu	75%	78%	80%	81%	82%	83%	83%	83%

Cell count examination revealed predominant mononuclear cells (75%); macroscopic parameters showed an exudate according to the criteria of yellow, semiclear, and clotty; furthermore, results of microbiologic tests for detection of bacteria, mycobacteria, and fungi were negative. The biochemical analysis demonstrated sugar, T‐protein, albumin, and LDH were 206, 4300, 2200, and 605, respectively. The SARS‐CoV‐2 RT‐PCR assay revealed the presence of the virus at the pleural fluid and not detected at nasopharyngeal swabs (Table 2). On the eighth day of hospitalization, despite the treatments, the patient's creatinine level did not improve significantly, and the patient died on August 8, 2020, due to a heart attack.

**TABLE 2:** Pleural fluid characteristics

Parameter	Results
Appearance	Semi‐clear
Color	Yellow
Total protein	4300 mg/dL
Albumin	2200 mg/dL
LDH	605 U/L
Glucose	206 mg/dL
WBC count	160/mm^3^(75% of mononucleated cells)
Microbiology	Negative
SARS‐CoV‐2 (RT‐PCR)	
Pleural fluid	Positive
Nasopharyngeal swab	Negative

### 2.2 Case 2: A 58‐year‐old man

A 58‐year‐old man with a history of liver transplant and dialysis (three times a week) was admitted to Fariman Hospital. The patient was admitted with symptoms such as fever and chills, headache, myalgia, and dyspnea. After ten days, he developed a left temporal headache and blurring of the left eye. Hence, the patient was referred to Imam Reza Hospital. The present patient had dyspnea, weakness, and lethargy, hypoxia, severe left temporal headache, nausea, and relative consciousness. In the early examinations, ptosis and proptosis of the left eye were spotted. The vision of the left eye is limited to counting fingers from a distance of two meters. The conjunctiva was a pale and weak macular reflex.

At the time of admission, blood pressure was 140/90 mm Hg, heart rate was 82 beats/minute, respiratory rate was 25/minute, body temperature was 37.5°C, and oxygen saturation of the room was 75%. Arterial blood gas has shown PH = 7.36 PO2=56 mm Hg, PCO2=32.3 mm Hg, and HCO3=18.6 meq/L. Primary laboratory tests showed blood sugar 129 mg/dL, calcium 6.7 mg/dL, phosphor 5.7 mg/dL, and magnesium. The urea and creatinine were 40 and 3.1, respectively. The blood sample revealed the following results: white blood cell count of 6100 cells per microliter with 6.7% lymphocytes, 89.3% neutrophils, and mildly elevated C‐reactive protein (CRP = 23.8 mg/L). On the third day of hospitalization, the patient developed GI bleeding in the form of coffee‐ground vomiting. Treatment was started with intravenous PPI and due to a drop in hemoglobin level on the 5th day of hospitalization, the patient received two units of packed cell during dialysis. The results of all biochemical tests are available in Table 3.

**TABLE 3:** The biochemical test during hospitalization

	1th day	2th day	3th day	4th day	5th day	6th day	7th day	8th day
WBC	6.1	9.5	17.2	13.3	12.2	13.3	13.1	13.5
Neut%	89.3%	96%	85%	82%	75%	80%	94.8%	83%
Lymphocyte count	6.7%	1.8%	4.1%	3.8%	3.3%	2.9%	2.1%	3.2%
Hemoglobin	11.5	11.5	12.1	8.6	7.9	12.4	10.3	10
Platelet count	16.7	61	94	86	74	65	53	50
Urea	105	114	113	128	135	159	205	200
Creatinine	4.5	4.7	4.4	4.8	5.1	2.8	5.9	5.8
Sodium	131	123	134	136	138	139	140	142
Potassium	4.4	4.4	4.3	4.4	4.6	4.4	5.2	5.4
O2 satu	75%	78%	80%	81%	82%	83%	83%	83%

**FIGURE 3:**
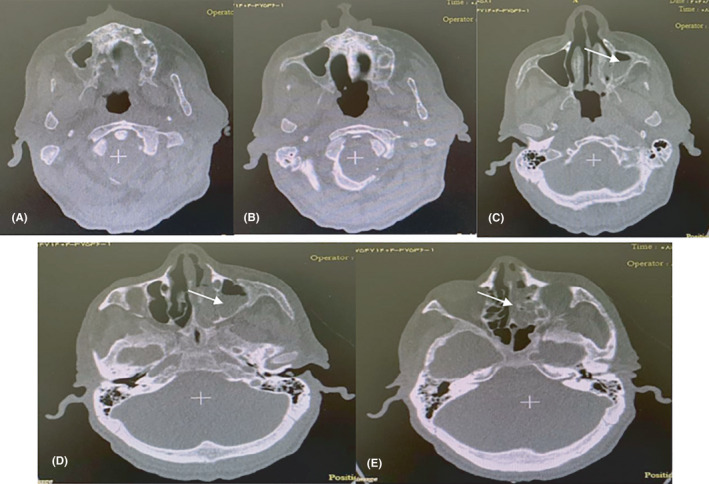
sinus CT Scan. On the first day of hospitalization pan‐sinusitis left sinuses (turbidity of maxillary and ethmoid left sinuses)

At Imam Reza hospital, blood investigation showed a white cell count increase, lymphocytes dominant, relative neutrophil decrease, and C‐reactive protein increase. Subsequent examinations included sinus CT demonstrating were shown turbidity of maxillary and ethmoid left sinuses (Figure 3). Diagnosis of pan‐sinusitis, evacuation sinusitis, and biopsy to rule out mucormycosis was performed.

**FIGURE 4:**
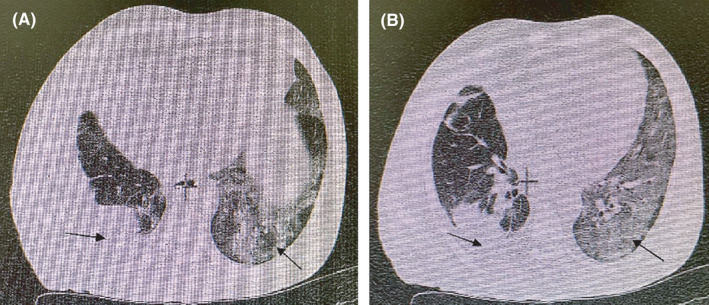
HRCT chest imaging. On the first day of hospitalization. A, Multifocal ground glass opacities in both lungs with more involvement of the left lung and large volume with a collapse of the lower lung on the right and B. multifocal ground glass opacities in both lungs with more involvement of the left lung and sporadic consolidation in the right lung

**FIGURE 5:**
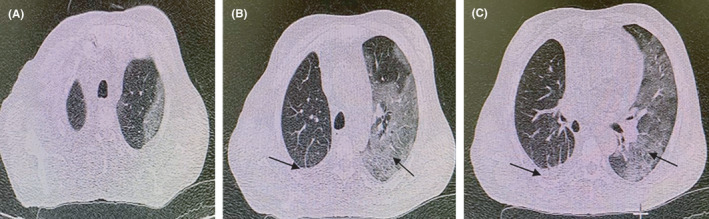
HRCT chest imaging. After four days of treatment. Relative reduction of pleural effusion, collapse of the lower lung on the right hemithorax, and sporadic consolidation and multifocal ground glass opacities on the left lung during management

The first chest CT showed disseminated ground glass opacity and sporadic consolidation in the right lung. The same symptoms were less severe in the right lung plus a moderate volume of pleural effusion (Figure 4). At the second chest CT, a little volume pleural effusion was observed at the base of the right hemithorax. The patient was transferred to the ICU with these symptoms (Figure 5). Based on radiographic results, 20 mL of clear yellow pleural fluid was removed, and the specimen was sent for differential macroscopic and microscopic analysis, cultures, biochemical examination, and SARS‐CoV‐2 RT‐PCR.

Cell count results illustrated predominant mononucleated cells (70%); macroscopic parameters indicated an exudate according to the criteria of yellow and semiclear; besides, results of microbiologic tests for detection of other pathogens such as bacteria, mycobacteria, and fungi were negative.

Biochemical analysis demonstrated that the levels of sugar, T‐protein, albumin, and LDH were 130, 2500, 1700, and 606, respectively. The SARS‐CoV‐2 RT‐PCR assay revealed the presence of the virus at the pleural fluid and nasopharyngeal swab (Table 4).

**TABLE 4:** Pleural fluid characteristics

Parameter	Results
Appearance	Semi‐clear
Color	Yellow
Total protein	2500 mg/dL
Albumin	1700 mg/dL
LDH	606 U/L
Glucose	130 mg/dL
WBC count	480/mm^3^(75% of mononucleated cells)
Microbiology	Negative
SARS‐CoV‐2 (RT‐PCR)	
Pleural fluid	Positive
Nasopharyngeal swab	Positive

During emergency treatment, intermittent noninvasive ventilation was considered and started intravenous (IV) Vancomycin 500 mg daily, Dexamethasone 4 mg bid, Famotidine 40 mg bid, and Amlodipine 5 mg daily. Also, Atorvastatin 40 mg and Remedicivir 100 mg daily, and amphotericin B 300 mg after hemodialysis on dialysis days were prescribed. Also, he underwent intravenous administration of Meropenem 500 mg bid and Enoxaparin 60 mg daily. In the first week, the patient received 12 million units of interferon every other day but was stopped due to the increase of liver enzymes. It should be considered that the dosage is not real as the patient was on dialysis, and the drug had been adjusted according to GFR.

## 3 DISCUSSION

Coronavirus 2019 (COVID‐19) is a beta‐coronavirus, similar to the acute coronavirus syndrome (SARS‐CoV) in 2003 but different from the monophyletic group.^1^ Respiratory symptoms are the most common and well‐studied clinical symptoms of this virus.^12^

The current diagnostic approach to COVID‐19 is mainly based on RT‐PCR positive for SARS‐CoV‐2 in nasopharyngeal swabs.^7^ The sensitivity of this method is limited; however, the virus can be detected in specimens from other sites, especially in later stages.^13^ For this reason, RT‐PCR has also been used on other biological materials, such as BAL fluids and feces.^14^ To the best of our knowledge, this is the first case of SARS‐CoV‐2 being detected in the pleural fluid in Iran.

The nasopharyngeal test of the two mentioned patients in this study was negative. In one of them, the pleural test was positive, which can be considered as a reliable test in the secondary stages. Therefore, recognition of a pleural effusion is also essential for optimizing the patient's prognosis, as fluid retention considerably improves respiratory dynamics and leads to better lung expansion (especially with supporting positive ventilation pressure). Another important message of these cases is the positive pleural fluid versus the negative results of the nasopharyngeal and oropharyngeal swabs, which can be helpful in the later stages of the disease. Pleural effusion is an uncommon symptom in COVID‐19 infection. Nevertheless, physicians should be aware of this potential symptom as the rapid diagnosis may be useful to optimize diagnostic evaluation in patients with upper respiratory tract RT‐PCR as well as the management of these patients.

## CONFLICT OF INTEREST

None to declare.

## AUTHOR CONTRIBUTION

ZA: compiled the endocrine data. SH: initiated the preparation of this case report for scientific publication. AM: coauthored and revised the manuscript and did proofreading. LA: searched the literature on this subject. SAJ: contributed substantially to the contents of the final version of this manuscript. All authors were directly or indirectly involved in the care of the patient.

## ETHICAL APPROVAL

Patient relative's consent was obtained for publication.

## Data Availability

Data presented in this manuscript are available upon request. Sented in this publication – analyzed clinical data and prepared the manuscript. AMK: provided medical consultation and received funding resources. RBM: provided medical consultation. NAC: involved in laboratory data collection and analysis and prepared the manuscript. EBB, MD: principal investigator – is involved in data analysis and manuscript preparation.
